# Gender Difference in Psychological, Cognitive, and Behavioral Patterns Among University Students During COVID-19: A Machine Learning Approach

**DOI:** 10.3389/fpsyg.2022.772870

**Published:** 2022-04-01

**Authors:** Yijun Zhao, Yi Ding, Yangqian Shen, Wei Liu

**Affiliations:** ^1^Computer and Information Sciences Department, Fordham University, New York, NY, United States; ^2^Graduate School of Education, Fordham University, New York, NY, United States

**Keywords:** COVID-19, gender difference, association rule mining, university student, mental health

## Abstract

The COVID-19 pandemic affects all population segments and is especially detrimental to university students because social interaction is critical for a rewarding campus life and valuable learning experiences. In particular, with the suspension of in-person activities and the adoption of virtual teaching modalities, university students face drastic changes in their physical activities, academic careers, and mental health. Our study applies a machine learning approach to explore the gender differences among U.S. university students in response to the global pandemic. Leveraging a proprietary survey dataset collected from 322 U.S. university students, we employ association rule mining (ARM) techniques to identify and compare psychological, cognitive, and behavioral patterns among male and female participants. To formulate our task under the conventional ARM framework, we model each unique question-answer pair of the survey questionnaire as a market basket item. Consequently, each participant's survey report is analogous to a customer's transaction on a collection of items. Our findings suggest that significant differences exist between the two gender groups in psychological distress and coping strategies. In addition, the two groups exhibit minor differences in cognitive patterns and consistent preventive behaviors. The identified gender differences could help professional institutions to facilitate customized advising or counseling for males and females in periods of unprecedented challenges.

## 1. Introduction

The COVID-19 pandemic has resulted in a devastating loss of human life worldwide and extraordinary challenges to public health (Knuppel, 2021). Besides directly affecting patients' well-being, indirect impacts have caused disruptions to many aspects of people's lives globally. Since March 2020, state and local authorities have taken steps to limit large gatherings and close non-essential businesses, triggering a near-total shutdown in many U.S. states.

University students have faced particular challenges during the COVID-19 pandemic. Many universities have suspended in-person activities and adopted virtual or hybrid teaching modalities (Gardner, 2020). Study-abroad programs have been shortened or suspended. Given the uncertainty of when normalcy will return, students are uneasy about the course of their academic careers. As a result, many students are facing unprecedented challenges, which can lead to a high level of psychological distress and changes in behavioral patterns. In addition, gender differences have been shown to exist after individuals are exposed to traumatic events (Street and Dardis, 2018). To respond to potentially traumatic events or life stressors, females are more vulnerable to developing mental health symptoms or physical problems than males (Yamada et al., 2018; Ishiguro et al., 2019; Vigna et al., 2019). Thus, in the study, we aimed to explore gender differences in psychological, cognitive, and behavioral patterns among undergraduate and graduate students during the COVID-19 pandemic.

Association rule mining (ARM) is a machine learning technique for discovering non-trivial, implicit, and useful relationships between the covariates of a dataset (Agrawal et al., 1993). ARM has emerged as one of the most popular techniques for pattern recognition in various application domains (Telikani et al., 2020), including COVID-19 related studies (Ren et al., 2020; Wu et al., 2020; Tandan et al., 2021). The goal of this study was to leverage a large dataset our team collected after the onset of the pandemic and apply machine learning techniques to discover psychological, cognitive, and behavioral patterns among U.S. university students when facing unprecedented challenges. In particular, we utilized association rule mining (Agrawal et al., 1993) to extract implication rules among the male and female participants, respectively. Based on those findings, we examined university students' gender differences in response to the COVID-19 pandemic. In our discussion, we compare our findings to related studies and discuss potential clinical implications of our study.

The contribution of this study is twofold. First, to the best of our knowledge, there is no existing work that applies ARM to studying gender differences in students' emotional and behavioral while coping with COVID-19. Our study aims to fill this gap by mining similar and distinct patterns among male and female university students at the peak of the pandemic. Second, although there have been studies examining individuals' psychological distress and changes in cognition and behaviors during COVID-19 (Wang et al., 2020; Ambelu et al., 2021; Bendau et al., 2021; Lorant et al., 2021), there is a lack of research examining how females and males differ in their psychological, cognitive, and behavioral patterns in response to the pandemic. Our study focuses on gender differences in the aforementioned domains among university students who face unprecedented challenges. Our findings can help professional institutions and community agencies develop multi-tiered (university-, school-, program-, and community-level) policies to improve students' emotional stability, self-control, and overall well-being during the pandemic.

## 2. Related Work

### 2.1. Psychological Distress

Government intervention to reduce the spread of the COVID-19 virus, including school closures, bans of social gathering and public events, and lockdowns, can have a non-negligible impact on the mental health of the general public (Qiu et al., 2020). Previous studies have shown that individuals may experience various mental health disorders including post-traumatic stress disorder (PTSD), depression, generalized anxiety disorder, panic disorder, and substance abuse due to isolation in managing infectious diseases (Norris, 2005; Mihashi et al., 2009; Jeong et al., 2016). In particular, suspension of social events and requirements of social distancing reduce opportunities for in-person interaction, which is an essential source of emotional support. The substantial changes in working conditions (e.g., increased workload for health professionals, telecommuting from home) can cause significant pressure and challenges in maintaining productivity. School closures have forced working parents into additional roles of full-time care givers for young children. Consequently, many individuals have experienced confined physical activities and increased screen time. The correlation between psychological distress and the COVID-19 pandemic has been examined in numerous studies (Wang et al., 2020; Ambelu et al., 2021; Bendau et al., 2021; Lorant et al., 2021).

### 2.2. Cognitive Patterns

According to the health belief model (HBM), individuals react to a health disease and decide whether to take action to prevent or control possible risks through their perceptions of both the susceptibility and severity of the condition and the benefits and barriers of behavioral changes (Champion and Skinner, 2008). The HBM is derived from psychological and behavior theory, suggesting that health-related behaviors are determined by individuals' desires to avoid risks and their beliefs about whether specific actions will prevent or reduce the risk of serious disease.

Perceived susceptibility refers to subjective perceptions of the risk of acquiring a disease, which is related to personal feelings about vulnerability to the risk. Perceived severity refers to subjective perceptions of the seriousness of contracting a disease and often involves considerations of possible medical consequences (e.g., disability, death) and social consequences (e.g., family functions, interpersonal relationships). If people view themselves as susceptible to a disease that could potentially lead to serious consequences, they are more likely to experience emotional reactions and take behavioral action to reduce their risks. Perceived benefits refer to perceptions of the effectiveness of available actions in diminishing the disease risk. Perceived barriers refer to perceptions of the obstacles to performing these actions. People's evaluations of the benefits and barriers vary greatly, leading to a cost/benefit analysis, which plays an important role in their reactions to a health condition. People are more likely to accept recommended health-related behaviors that are perceived as beneficial and that have fewer barriers.

The HBM has been widely applied to understanding people's adherence to disease prevention and treatment as well as developing interventions for improving adherence (Jones et al., 2014). Many recent studies focusing on the COVID-19 health crisis have also applied HBM to predict the likelihood that people will adopt certain preventive behaviors, including wearing masks, maintaining social distance, and receiving vaccines (Tong et al., 2020; Wong et al., 2020, 2021; Shmueli, 2021).

### 2.3. Behavioral Patterns

#### 2.3.1. Preventative Behaviors

Suggested by the HBM, people's preventive behaviors are typically determined by their perceived susceptibility and severity and perceived benefits and barriers. In response to authority-imposed measures, individuals exhibit different levels of compliance governed by their cognitive patterns under specific conditions. Recent studies examining individuals' behavioral response to the COVID-19 have also indicated the variance in people's preventative behaviors.

#### 2.3.2. Communication

Additionally, people tend to demonstrate different patterns of communication regarding the COVID-19 pandemic, which have exposed people to an array of challenges, including psychological distress, anxiety, fatigue, fear, and social isolation. Effective communication has become a critical barrier in the workplace, communities, and families. Communication is defined as sending or exchanging messages through the modalities of speaking, writing, or other types of media (Reddy and Gupta, 2020). During peaks in the pandemic, social media, the internet, and social networking have generated a deluge of information about COVID-19. Although individuals have largely relied on recommendations by local governments or central administration agencies such as the Centers for Disease Control (CDC) to regulate their behavior and expectations, fear, distrust, and resistance have also been common reactions to different types of response measures suggested by authoritative sources. In addition to public information, credible information obtained and confirmed through communication with friends and family members has been critical for motivating people to take action.

### 2.4. Gender Differences

The effects of increasing psychological distress and changes in cognitive and behavioral patterns in response to COVID-19 can vary among individuals due to different demographic factors. Gender is one important factor for such variance. In research by Yan et al. (2021), a survey of 1,749 females and 1,339 males in China revealed that females and males differed significantly in their adjustment to living/working conditions, responses to having a fever, and the need for psychological support during the COVID-19 outbreak. Similarly, Ştefănuţ et al. (2021) reported that levels of depression, anxiety, and stress were significantly higher in females than in males in response to the COVID-19 pandemic. These empirical findings indicate the importance of examining gender differences in emotional, cognitive, and behavioral responses to the COVID-19 outbreak in the United States. Many recent studies examined the gender differences among the general population. However, undergraduate and graduate students are going through a unique stage of life experiences, such as leaving their families to develop more independence and living in a dormitory environment that is quite different from a home environment. Thus, the gender differences among undergraduate and graduate students warrant further examination.

### 2.5. The Impact of COVID-19 on Undergraduate and Graduate Population

School closures reduce social contact among students, faculty, and staff, and therefore interrupt transmission of disease. A 2018 metanalysis of 25 studies examining school closures found that implementing school closures before or after an epidemic reached its peak reduced overall influenza spread. Earlier school closures in history reduced and delayed epidemic peaks, and longer closures resulted in delayed epidemic peaks (Nafisah et al., 2018). By the end of spring 2020, over 4,234 colleges and universities across the United States had been impacted by COVID-19 (Entangled Solutions, 2020).

Like the rest of the population, university students experienced the psychological effects of social distancing. Social distancing precautionary measures resulted in people experiencing separation from loved ones, loss of freedom, boredom, and hyper-uncertainty over disease status (Brooks et al., 2020). A 2020 metanalysis examining quarantining during previous epidemics worldwide (e.g., North America, Asia, Africa, Australia) highlighted the negative effects of social isolation, including post-traumatic stress symptoms, confusion, and anger. Stressors during quarantine include duration of quarantine, fears of infection, frustration, boredom, and inadequate supplies and information. In short, it is critical to examine undergraduate and graduate students in the areas of psychological, cognitive, and behavioral characteristics in response to the impact of COVID-19.

## 3. Materials and Methods

### 3.1. Participants and Procedures

This research was approved by the Institute Review Board of Fordham University. We recruited the participants through email broadcasting, posts on social media such as Facebook or Instagram, and announcements through some student organizations. All participants were provided informed consent, which noted the confidentiality rules and their voluntary participation in this study. There were some eligibility criteria, including all participants must be at least 18 years old and physically reside in the United States when they completed the questionnaires. Furthermore, all participants should enroll in an undergraduate or graduate program. The questionnaire took about 10–15 min to complete, and an incentive was offered to participants for completing the questionnaires (e.g., special postcards or gift cards through raffles).

All data were collected from May 2020 to July 2020, a peak period of COVID-19. During this period, most public school systems in the United States were mandated to conduct virtual schooling, and many universities in the United States also resorted to fully virtual classrooms. Within the educational systems, large social gatherings, athletic events, and even in-person graduation commencement were temporarily suspended. In addition, the United States placed air-travel restrictions on locations where COVID-19 outbreaks had occurred (Clarke, 2020; White House, 2020). International travelers were asked to self-quarantine for 14 days (CDC, 2020).

A total of 366 US university students were recruited and administered a set of self-report questionnaires through Qualtrics. Data quality control was performed by manually examining all survey reports to ensure participant eligibility and no excessive missing answers in each report. We excluded 37 participants who reported being outside the US at the survey time. Furthermore, we recognized that there were more than two gender categories. However, for this study, we excluded seven survey reports rendered by non-binary gender participants due to their limited sample size. The final data for our analysis consisted of 322 male and female university students.

Table 1 presents the demographic characteristics of our dataset. Approximately 28.6% of the 322 participants reported residency within the Metropolitan New York area, a COVID-19 epicenter during the early stage of the pandemic. In addition, the dataset consisted of an equal number of undergraduate and graduate students (161, respectively) and an approximate male to female ratio of 1:7. It is worth noting that using a convenient sample through authors' professional networks could have caused the disproportion between the two gender groups. Specifically, students in psychology and social science fields were largely recruited, leading to more female participants. Nevertheless, according to our experimental results, we were able to obtain a sufficient number of meaningful patterns for the male group (**Table 4**) and conduct a valid comparison to the findings of the female group.

**Table 1:** Demographic characteristics of participants (*N* = 322).

**Variable**	***N***	**%**
Student status		
Undergraduate	161	50%
Graduate	161	50%
Location during the peak of COVID-19		
NYC	92	28.6
Outside of NYC	230	71.4
Gender		
Male	41	12.7
Female	281	87.3

### 3.2. Measures

#### 3.2.1. Perceived Susceptibility and Perceived Severity

The questionnaires used to assess perceived susceptibility and perceived severity of COVID-19 were developed based on the health belief model (Champion and Skinner, 2008). There were four items for each variable and all items (e.g., “my chances of getting COVID-19 are high”) used a 5-point Likert scale, ranging from 1 (strongly disagree) to 5 (strongly agree). Higher scores indicated a higher level of perceived susceptibility/severity in relation to COVID-19. The questionnaires demonstrated good reliability and validity in the present study [perceived susceptibility: Cronbach's α = 0.76, composite reliability (CR) = 0.85, average variance extracted (AVE) = 0.60; perceived severity: Cronbach's α = 0.77, CR = 0.85, AVE = 0.60].

#### 3.2.2. Perceived Benefits and Perceived Barriers

The questionnaires used to assess perceived benefits and perceived barriers of staying at home during the peaks of COVID-19 were developed based on the measure that was used to assess outcomes in implementing SARS-preventive behaviors (Cheng and Ng, 2006). The items specifically in relation to benefits and barriers of preventive behaviors were extracted and adapted to fit the context of COVID-19. There were four items for each variable, and all items (e.g., “staying at home prevents me from getting COVID-19”) used a 5-point Likert scale, ranging from 1 (strongly disagree) to 5 (strongly agree). Higher scores indicated a higher level of perceived susceptibility/severity in relation to COVID-19. The questionnaires demonstrated good reliability and validity in the present study (perceived benefits: Cronbach's α = 0.79, CR = 0.86, AVE = 0.62; perceived barriers: Cronbach's α = 0.90, CR = 0.93, AVE = 0.71).

#### 3.2.3. Psychological Distress

In the domain of psychological distress, 10 questions were developed based on Gross and John (2003). The questions consisted of items such as, “During the peak time of COVID-19, I felt depressed.” All items used a 5-point Likert scale, ranging from 1 (strongly disagree) to 5 (strongly agree). Higher scores indicated higher levels of endorsement of psychological distress. The questionnaires demonstrated good reliability and validity in the present study (Cronbach's α = 0.91, CR = 0.92, AVE = 0.55).

#### 3.2.4. Communication

In the domain of communication, 13 questions were developed based on Morton and Duck (2001). The items mainly included three aspects of communication, including communication through interpersonal relationships (e.g., with peers, families, healthcare professionals, colleagues) and public media (e.g., television, newspaper, magazine, social media such as Facebook, official and unofficial websites), and how much attention was directed to such communication. There were five questions regarding communication through interpersonal relationships that used a 5-point Likert scale, ranging from 1 (not at all) to 5 (a great deal). There were seven questions regarding communication through public media that used a 5-point Likert scale, ranging from 1 (never) to 5 (always). One question regarding whether the participant paid attention to news and information regarding COVID-19 in the media used a 5-point Likert scale, ranging from 1 (strongly disagree) to 5 (strongly agree). The questions consisted of items such as “How much have you discussed COVID-19 with the following people during the peak time of COVID-19?” The questionnaires demonstrated good reliability and validity in the present study (Cronbach's α = 0.80, CR = 0.85, AVE = 0.31).

#### 3.2.5. Preventative Behavior

The questionnaire used to assess participants' preventative behaviors was originally designed by the team at Beijing Normal University. Given the unique nature of COVID-19, the government imposed a series of prevention behaviors, including staying at home, wearing masks, washing hands, cleaning surfaces, and maintaining social distance, which was the main focus of this questionnaire. Some sample questions included, “How frequently did you go outside during the peak time of COVID-19?”, “How frequently did you wash your hands during the peak time of COVID-19?”, and “How frequently did you maintain social distance during the peak time of COVID-19?” A 7-point Likert scale was used for this questionnaire ranging from 1 to 7. The questions were initially developed based on previous health crisis-related research with modifications to fit the situations applicable to COVID-19.

### 3.3. Methods

#### 3.3.1. Association Rule Mining

Association rule mining (ARM) is widely used in market basket analysis, which provides retailers with non-trivial, implicit, and previously unknown information to understand customers' purchase patterns. The uncovered relationships are represented in the form of “association rules” extracted from a retail store's transaction database, such as {Diaper} → {Beer} (Zhao and Bhowmick, 2003). This rule suggests that a strong relationship exists between the sale of diapers and beer in one transaction (i.e., many customers who bought diapers also bought beer). Consequently, retailers can benefit from this type of rule to identify new opportunities such as cross-selling or product shelf arrangements. Formally, let


$$I=\left\{i_{1},i_{2},\ldots i_{d}\right\}$$

be the set of all distinct items in a market basket data and


$$T=\left\{t_{1},t_{2},\ldots t_{N}\right\}$$

be the set all customer transactions. Each transaction *t*_*i*_ (*i* = 1, 2, …*N*) contains a subset of items from *I*. We define an itemset *U* to be a collection of items, i.e.,


$$A=\left\{a_{1},a_{2},\ldots a_{k}\right\}$$

where *a*_*i*_∈*I* ∀*i*∈[1, *k*] and |*A*| ≤ *d*.

An association implication takes the form *X*→*Y* where both *X* and *Y* are itemsets. Given a set of transactions *T*, the overarching goal is to find all association rules with non-negligible coverage and strong implications. The prevalence is measured by *support* which is defined as the fraction of transactions that contain both *X* and *Y* among the total |*T*| transactions. The implication strength is defined by *confidence* which measures how often items in *Y* appears in transactions that contain *X* i.e.,


$$confidence\left(r\right)=\frac{s\left(X,Y\right)}{s\left(X\right)}$$

where *s* denotes the *support*. In practice, two thresholds *minsup* and *minconf* are employed to identify strong association rules with support and confidence above the *minsup* and *minconf*, respectively.

In addition to market basket analysis, ARM is employed in other application areas including web usage mining (Asadianfam et al., 2020), medical diagnosis (Sarıyer and Öcal Taşar, 2020), intrusion detection (Safara et al., 2020), customer relationship management (Gangurde et al., 2020), and bioinformatics (Ceddia et al., 2020).

#### 3.3.2. Lift

ARM algorithms rely on the minimum *support* and *confidence* thresholds to select interesting patterns. One limitation of this framework is that an identified association rule can be misleading even if its *support* and *confidence* are above the *minsup* and *minconf* thresholds. For example, Table 2 summarizes a hypothetical dataset of 1000 students to study the impact of a mentor's feedback type (positive or negative) on a student's creativity in research. Any rule with a *support* above 10% and a *confidence* above 70% is arguably a strong association rule. Thus, using *minsup* = 10% and *minconf* = 70% as model parameters, an ARM algorithm will extract *r*1: {Negative Feedback} → {Creative} as one of the behavioral patterns due to its high *support* (15%) and *confidence* (75%) levels. Nevertheless, we observe that the expected percentage of creative students in the entire study cohort is 90%, while only 75% of students are creative among those who received negative feedback. Thus, negative feedback indeed has a negative association with being creative.

**Table 2:** Feedback type and student creativity statistics.

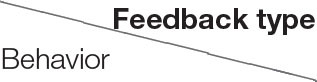	**Negative feedback**	**Positive feedback**	**Total**
Creative	150	750	900
Not creative	50	50	100
Total	200	800	1,000

One approach to address the above limitation is to further filter the generated strong association rules using *lift*. Given an association rule *r*: *X*→*Y*, the *lift* of *r* is defined as:


$$lift\left(r\right)=\frac{c\left(X\to Y\right)}{s\left(Y\right)}=\frac{s\left(X,Y\right)}{s\left(X\right)s\left(Y\right)}$$

where *s* and *c* denote *support* and *confidence*, respectively. Consequently,


$$lift(r)=\left\{ \begin{array}{l} =1\text{ if }X\text{ and }Y\text{ are independent}\\ >1\text{ if }X\text{ and }Y\text{ are positively correlated }\\ <1\text{ if }X\text{ and }Y\text{ are negatively correlated} \end{array}\right.$$

and we are only interested in rules with *lift* >1. In the above example, although *r*1 is a strong association rule, it will not be retained because *lift* ({Negative Feedback} → {Creative}) = 0.15/(0.2*0.9) = 0.8333, indicating a negative correlation. In our study, we require *lift* >1.2 to ensure the validity of the extracted behavioral patterns.

### 3.4. Encoding Q&As as Market Basket Items

We employed association rule mining techniques to our proprietary survey data to study gender dissimilarities in the various psychological domains. To formulate our task under the conventional market basket analysis framework, we modeled each unique question-answer pair in the survey questionnaire as a market basket item. We viewed participants as customers, and their survey reports were consequently analogous to customer transactions on a collection of items.

Table 3 illustrates our encoding method to transform question-answer pairs into market basket items. Since all questions in our survey have less than ten answer choices, we represented each unique question-answer pair by concatenating a question's number and a corresponding answer index as one item. That is, the last digit of an item code specifies the answer index and the number proceeding “-” indicates the question number. As an example, Question 153 states “During the peak time of COVID-19, I felt worthless.”, and there are five choices for this question. Consequently, five unique items (“153-1”, “153-2”, “153-3”, “153-4”, and “153-5”) were generated from this question, each of which the number 153 specifies the question index and the last digit (1–5) indicates the five answers, respectively. Following this approach, each question contributed *k* unique items to the market basket where *k* < 10 is the number of answer choices for the question.

**Table 3:** Survey question-answer encoding.

**Question #**	**Question**	**Answer choices**	Items^*^
1	During the semester of Spring 2020, you were a(n)	0. Not a student 1. Undergraduate 2. Graduate	1-0 1-1 1-2
2	Your gender	0. Male 1. Female 2. Other	2-0 2-1 2-2
	⋮	⋮	⋮
37	I am more likely than the average person to get COVID-19.	1. Strongly Disagree 2. Disagree 3. Neutral 4. Agree 5. Strongly Agree	37-1 37-2 37-3 37-4 37-5
	⋮	⋮	⋮
41	During the peak time of COVID-19, I kept my emotions to myself.	1. Strongly Disagree 2. Disagree 3. Slightly Disagree 4. Neutural 5. Slightly Agree 6. Agree 7. Strongly Agree	41-1 41-2 41-3 41-4 41-5 41-6 41-7
	⋮	⋮	⋮
153	During the peak time of COVID-19, I felt worthless.	1. Strongly Disagree 2. Disagree 3. Neutral 4. Agree 5. Strongly Agree	153-1 153-2 153-3 153-4 153-5

* *Each question-answer combination contribute to one market basket item. Last digit of an item specifies the answer index. The number proceeding ‘-' indicates the question number. Each question contributed k unique items to the market basket where k < 10 is the number of answer choices for the question*.

### 3.5. Frequent Pattern Generation

**Figure 1:**
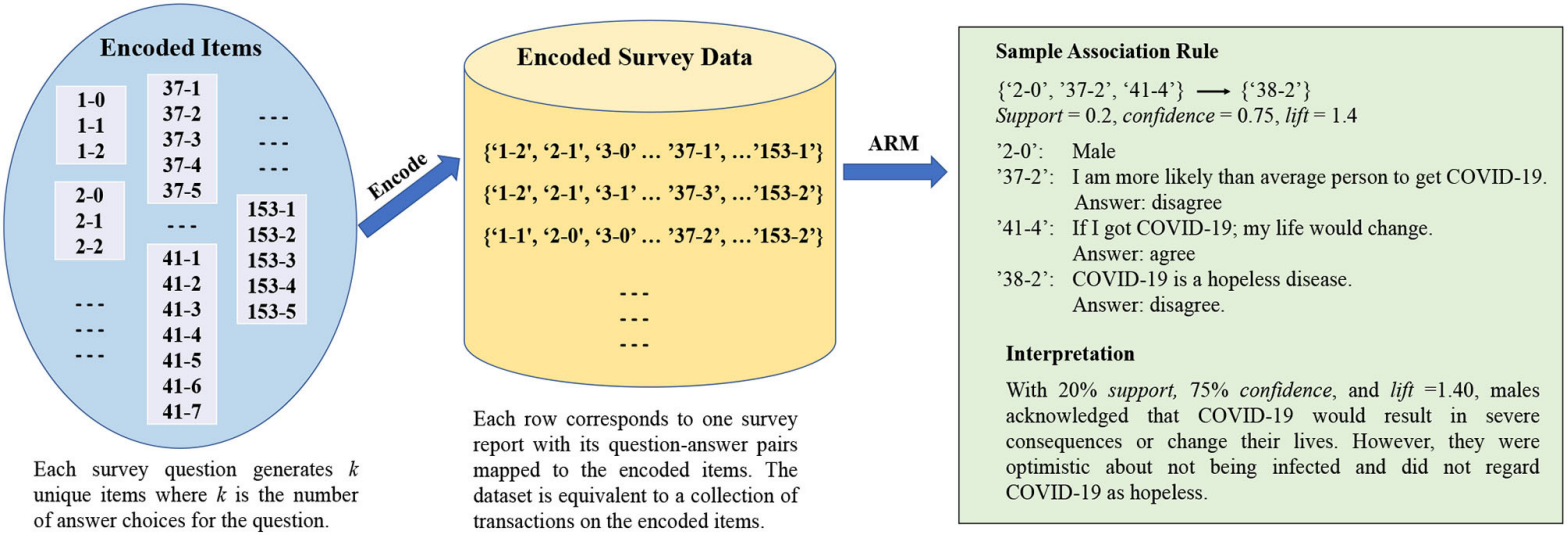
Association rules generation from survey data.

Figure 1 presents the pipeline of our frequent pattern generation process. First, all unique items were generated (left component) using the above Q&A encoding system. A sample itemset could take the form of “2-0”, “37-2”, “41-4”, which indicates the individual selected “2” for Question 37, “0” for Question 2, and “4” for Question 41. Next, survey reports were modeled as itemsets purchased by customers (i.e., participants), with the question-answer pairs mapped to the encoded items. As a result, the entire dataset (middle component) is equivalent to a set of transactions on market basket items. Finally, we applied association rule mining techniques to extract all strong frequent patterns (right component).

To mine the strong implication rules in our data, we employed the frequent pattern growth FP-growth algorithm (Tan et al., 2006), which compresses the input data into a tree structure and recursively extracts the frequent patterns. FP-growth was selected due to its efficiency and scalability compared to other ARM algorithms, such as the Apriori algorithm (Inokuchi et al., 2000). We implemented the algorithm using the Python programming language (Van Rossum, 2007) and mined our strong association rules using 20 and 70% as the minimum *support* and *confidence* thresholds, respectively. The discovered rules were further filtered by requiring the interest factor (i.e., *lift*) above 1.20. For example, a sample association rule produced by our algorithm was as follows:

{‘2-0', ‘37-2', ‘41-4'} → {‘38-2'}, *support* = 0.2, *confidence* = 0.75, *lift* = 1.4

where

“2-0”: I am a male

“37-2”: I am more likely than average person to get COVID-19. Answer: disagree

“41-4”: If I got COVID-19; my life would change. Answer: agree

“38-2”: COVID-19 is a hopeless disease. Answer: disagree

The rule indicated that, with 20% *support*, 75% *confidence*, and *lift* = 1.40, males acknowledged that COVID-19 would result in severe consequences or change their lives. However, they were optimistic about not being infected and did not regard COVID-19 as hopeless.

Our findings suggested that significant differences existed for the two gender groups in participants' psychological distress levels and coping strategies. In addition, the two groups exhibited minor differences in cognitive patterns and consistent preventive behaviors. Details are provided in the Results and Discussion sections.

### 3.6. Quantitative Evaluation

In addition to examining the individual association rules, comparing the total number of rules extracted for each gender group in each psychological category can also provide insightful information. In particular, a larger number of discovered rules indicates more consistent behaviors in a category. Furthermore, the quantitative difference of rule numbers between the two gender groups for a given category signifies their degree of behavioral dissimilarities. Therein, we computed both the difference and the ratio of the total number of rules for each psychological category between the two groups. The difference was calculated as the number of rules for the male group minus that for the female group. The ratio was define as:


$$\text{ratio}=\frac{\max\left(M,F\right)}{\min\left(M,F\right)}$$

where *M* and *F* denote the numbers of rules for the male and female group, respectively.

## 4. Results

In this section, we present our main results in studying the behavioral differences between male and female university students' responses to the COVID-19 pandemic. We first examine the quantitative differences in the number of rules discovered for the two gender groups in each category. We then investigate specific different and similar behavioral patterns.

### 4.1. Quantitative Comparison of Implication Rules

Table 4 presents the total number of strong association rules discovered in the male and female groups across the five psychological categories in our survey. Columns “Diff” and “Ratio” are the two evaluation metrics defined in Section 3.6.

**Table 4:** Comparison of total association rules between gender groups.

**Index**	**Category**	**Male**	**Female**	**Diff**	**Ratio**
1	Communication	4	104	−100	26.00
2	Psychological distress	186	13	173	14.31
3	Perceived susceptibility and severity	16	3	13	5.33
4	Perceived benefits and barriers	36	106	−70	2.94
5	Preventive behaviors	22	14	8	1.57

We observe that the two gender groups demonstrated significant differences in Categories 1 and 2, with a difference ratio of 26 and 14.31, respectively. In Categories 3 and 4, males and females exhibited noticeable differences with a difference ratio of 5.33 and 2.49, respectively. For the “Preventive Behaviors” category, the difference was less salient (i.e., ratio = 1.57). Thus, we expected the most interesting findings to occur in the first two categories.

### 4.2. Psychological Patterns

The two gender groups displayed significant differences in psychological distress level and coping strategies. Table 4 shows that the male group outnumbered the female group in the total number of rules (186 vs. 13) with a ratio of 14.31. Thus, males exhibited more consistent behavior than females in this category. Furthermore, we observed that males were more optimistic and resilient when facing extraordinary challenges evidenced by the following findings, which were not present in the female groups:

- Males agreed that they felt down, but they disagreed that “nothing could calm me down.”
- Males agreed that everything took effort, but they disagreed that they felt so restless they could not sit still.
- Males agreed that they felt nervous, but they disagreed that they felt hopeless, uncontrollable, or worthless.

### 4.3. Cognitive Patterns

Both cohorts showed very few extracted patterns (Female:3; Male:13) in perceived susceptibility and severity regarding the pandemic. Among the 13 male rules, we found that males acknowledged their worries about COVID-19 but were optimistic. Furthermore, males acknowledged that COVID-19 could result in severe consequences or change their lives. However, they were optimistic about not being infected and did not consider the pandemic as hopeless. Our findings also suggested that males perceived that they were safe staying at home but that doing so affected their schedule and daily activities. This pattern was not observed among the female participants.

### 4.4. Behavioral Patterns

Males and females showed consistent preventive behaviors in coping with the pandemic, that is, those who seldom went outside during the peak time of COVID-19 always followed the preventive behaviors advised by authorities. Furthermore, females exhibited more consistent behavior than males in establishing their communication networks, evidenced by the number of rules (i.e., female:104; male:4) and their ratio of 26. A closer examination of individual implication rules revealed that females tended to reach out to a greater variety of resources compared to males. For example, males who visited official university websites for updates were also likely to visit social media websites. For females, in addition to visiting social media websites, also shared information by talking to family members and peers. These interpersonal communication patterns were not present in the male cohort.

## 5. Discussion

### 5.1. Psychological Pattern

The findings suggest that there were noticeable differences in psychological distress level and coping strategies between male and female university students in response to the COVID-19 pandemic. Overall, males were more optimistic and resilient when facing extraordinary challenges evidenced in a number of areas. Our findings concur with previous research, suggesting that females tend to be more vulnerable to developing mental health problems or physical problems in response to stressful life circumstances or traumatic events (Yamada et al., 2018; Ishiguro et al., 2019; Vigna et al., 2019). Similar findings have been reported regarding psychological responses in relation to COVID-19, with more female medical workers reporting more negative responses associated with pandemic-related challenges (Zhu et al., 2020) and a higher prevalence of anxiety disorders being reported by women than by men (Swami et al., 2020; Yan et al., 2021). This suggests that males and females differ considerably in adapting to working/living conditions, symptoms of illness, and the need for psychological services. Our findings further support the need to provide mental health service to individuals with consideration of gender differences.

### 5.2. Cognitive Pattern

People's perceptions of susceptibility to and severity of health conditions play a key role in deciding whether to engage in behaviors that reduce their risks. However, certain self-reinforcing biases that may be related to gender differences can prevent accurate knowledge of susceptibility, resulting in some people feeling invulnerable and some people feeling hopeless in the face of a serious health crisis (Moussaoui et al., 2020). Our findings support this argument in that, compared to females, males acknowledged their worries about COVID-19 but were more positive about it. Galasso et al. (2020) examined the perceived threats and fear of COVID-19 based on a sample of 21,649 participants, concluding that females were more likely to perceive the COVID-19 pandemic as a severe issue, which was confirmed by Nino et al. (2021) based on a sample of 7,441 U.S. adults. Our findings reinforce previous research by showing that males recognized that COVID-19 could result in serious consequences or change their lives, but they were optimistic about not considering COVID-19 as hopeless. Additionally, males demonstrated a pattern of perceiving both high benefits and high barriers for following preventive behaviors at the same time, which was not found in females. One explanation for this discrepancy is that males of all ages are more physically active than females (Azevedo et al., 2007), resulting in more barriers for males to stay at home.

### 5.3. Behavioral Pattern

In the area of communication, females in the present study exhibited more consistent behavior than males, and females explored more interpersonal communication approaches (e.g., with family members) than males. During the COVID-19 pandemic, people might be stereotyped, discriminated against, or suffer due to perceived contact with COVID-19. Day-to-day behaviors (e.g., travel bans, quarantine requirements) might revolve around communication related to the pandemic. Effective communication about the pandemic might benefit the public, such as information about the physical and psychological well-being of themselves and their family members (Back et al., 2020; Dalton et al., 2020). Given that women tend to present more severe symptoms of anxiety, depression, and acute stress during the pandemic (Garćıa-Fernández et al., 2021), it is not surprising that women also present different communication patterns, which were revealed in the present study. It is plausible that males and females rely on different sources of communication to confirm their interpretation of the circumstances associated with COVID-19 and, thus, they differ in the sources of information they rely on and the need for interpersonal communication.

In recent studies that examined preventative behaviors during the COVID-19 pandemic, females were found to exhibit higher levels of precautionary behaviors guided by public policy from the government, such as washing hands, social distancing, non-utilization of public transport, and stay-at-home restrictions (Galasso et al., 2020; Guzek et al., 2020; Levkovich and Shinan-Altman, 2021; Zysset et al., 2021). However, our findings indicated that both males and females showed consistent preventive behavior patterns in coping with the pandemic. It may be the nature of human behavior that both men and women have similar patterns of vigilance and enforcement of necessary self-prevention in the face of a significant risky situation.

### 5.4. The Impact of COVID-19 on Undergraduate and Graduate Students

A survey of 2,086 students by Active Minds Inc., a national non-profit supporting mental health awareness and education for students, found that 80% surveyed reported that the pandemic negatively affected their mental health, with one in five students stating that during the COVID-19 pandemic, their mental health significantly worsened (Kerr, 2020; Minds, 2020). Additional challenges faced by university students during the pandemic related to (lack of) independence, being back home for an extended and unexpected period of time, social isolation, and high conflicts and tension (Brooks et al., 2020; Hess, 2020; Kerr, 2020). Our findings suggest that significant differences exist between the two gender groups among undergraduate and graduate students in psychological distress and coping strategies. The two groups exhibit minor differences in cognitive patterns and consistent preventive behaviors. The findings suggest that mental health counseling services and workshops focusing on stress management might be particularly important to high education students. Gender differences manifested through psychological, cognitive, and behavioral aspects need to be addressed to provide customized prevention and treatments.

### 5.5. Limitations and Future Research

Although our study demonstrates interesting gender differences and the effectiveness of the ARM algorithm, our dataset was limited to university students who were self-motivated to participate in the survey. Thus, our findings could be affected by a selection bias. We foresee one potential future study is to apply the same machine learning technique to validate our findings in a more general population.

Another limitation of this study involved the metrics used to assess participants' responses. It is worth noting that given COVID-19's unprecedented challenges, its unique nature, and the rigorous preventive rules imposed by the public health officials, there were no existing questionnaires that fit our research purpose. Thus, we modified the questionnaires in previous health crises-related research with additional self-designed questions. The quantitative measures employed in the questionnaires have not been established in classical psychometric publications. Nevertheless, we believe our metrics were proper and sufficient, evidenced by the consistency between our findings and existing psychological theories.

In addition, our findings indicated that males were more resilient than females during the pandemic, and females tended to report more adverse effects from the stress. In reality, men tend to be more self-reliant and internalize their distress, leading to fewer externalized behavioral symptoms. Some males might not realize that they were experiencing emotional distress and might not actively report such symptoms. Thus, the level of effectiveness for the machine learning approach to differentiate the gender difference warrants further examination.

Finally, this study mostly recruited individuals attending undergraduate or graduate schools, and the participants represented those who have received a high level of education in the United States. Therefore, the generalization of the findings might be limited. Future studies would need to recruit participants from more diverse educational and socio-economic backgrounds.

### 5.6. Clinical Implications

Our study is a novel approach that applies the ARM technique to explore gender differences through the perspectives of psychological, cognitive, and behavioral patterns in response to the COVID-19 pandemic. Our findings underscore the importance of paying attention to the mental health of the general public during the pandemic, especially those with higher risk of psychological distress (e.g., women, older individuals). The identified gender differences among participating students could help professional institutions to facilitate customized advising or counseling for males and females in periods of unprecedented challenges. In addition, a machine learning approach could be used in the blended approach (i.e., combining mental health counseling and computer science) and could be used as an assistive tool for screening and tracking distress.

## Data Availability Statement

The datasets presented in this article are not readily available because data will be provided to qualified investigators upon reasonable request. Requests to access the datasets should be directed to YD, yding4@fordham.edu.

## Ethics Statement

The studies involving human participants were reviewed and approved by the Institute Review Board of Fordham University. The patients/participants provided their written informed consent to participate in this study.

## Author Contributions

YZ and YD: conceptualization, methodology, resources, supervision, project administration, and funding acquisition. YZ and WL: software and data curation. YZ, YD, and YS: validation, formal analysis, writing original draft preparation, and writing review and editing. All authors have read and agreed to the published version of the manuscript.

## Funding

This project was funded by 2021-2022 Fordham University Interdisciplinary Research Grant awarded to YZ and YD and 2020 Fordham University COVID-19 Research Grant awarded to YD.

## Conflict of Interest

The authors declare that the research was conducted in the absence of any commercial or financial relationships that could be construed as a potential conflict of interest.

## Publisher's Note

All claims expressed in this article are solely those of the authors and do not necessarily represent those of their affiliated organizations, or those of the publisher, the editors and the reviewers. Any product that may be evaluated in this article, or claim that may be made by its manufacturer, is not guaranteed or endorsed by the publisher.
